# Elovanoids are novel cell-specific lipid mediators necessary for neuroprotective signaling for photoreceptor cell integrity

**DOI:** 10.1038/s41598-017-05433-7

**Published:** 2017-07-13

**Authors:** Bokkyoo Jun, Pranab K. Mukherjee, Aram Asatryan, Marie-Audrey Kautzmann, Jessica Heap, William C. Gordon, Surjyadipta Bhattacharjee, Rong Yang, Nicos A. Petasis, Nicolas G. Bazan

**Affiliations:** 1 Neuroscience Center of Excellence, School of Medicine, Louisiana State University Health New Orleans, New Orleans, LA USA; 2 Department of Chemistry and Loker Hydrocarbon Research Institute, University of Southern California, Los Angeles, CA USA

## Abstract

Docosahexaenoic acid (DHA, 22:6 n-3) is abundant in the retina and is enzymatically converted into pro-homeostatic docosanoids. The DHA- or eicosapentaenoic acid (EPA)-derived 26 carbon fatty acid is a substrate of elongase ELOVL4, which is expressed in photoreceptor cells and generates very long chain (≥C28) polyunsaturated fatty acids including n-3 (VLC-PUFAs,n-3). While ELOVL4 mutations are linked to vision loss and neuronal dysfunctions, the roles of VLC-PUFAs remain unknown. Here we report a novel class of lipid mediators biosynthesized in human retinal pigment epithelial (RPE) cells that are oxygenated derivatives of VLC-PUFAs,n-3; we termed these mediators elovanoids (ELV). ELVs have structures reminiscent of docosanoids but with different physicochemical properties and alternatively-regulated biosynthetic pathways. The structures, stereochemistry, and bioactivity of ELVs were determined using synthetic materials produced by stereo-controlled chemical synthesis. ELVs enhance expression of pro-survival proteins in cells undergoing uncompensated oxidative stress. Our findings unveil a novel autocrine/paracrine pro-homeostatic RPE cell signaling that aims to sustain photoreceptor cell integrity and reveal potential therapeutic targets for retinal degenerations.

## Introduction

Disease onset and progression trigger a complex cellular response that disrupts homeostasis^1, 2^. Referred to as inflammation, this is a defensive mechanism that includes the generation of protective mediators, including bioactive lipids^3–7^, and engages immune cells, blood vessels, neurons, astrocytes, retinal pigment epithelial (RPE) cells and other cells, aiming to sustain homeostasis, remove triggering factors and cell debris, and set in motion cellular and tissue restoration. Pro-homeostatic signaling is set in motion in RPE cells, photoreceptor cells (PRCs) and, likely, in other retinal cells at the beginning of cellular disruptions such as uncompensated oxidative stress (UOS), as well as at the onset of retinal degenerations^8–10^ or other neurodegenerative diseases.

The omega-3 fatty acid docosahexaenoic acid (DHA) is abundant in the central nervous system (CNS), which includes the retina^5, 6, 9, 11^, and serves as the precursor for 22-carbon chain length docosanoids, which have neuroprotective and pro-homeostatic bioactivities^9, 10, 12, 13^. DHA also can be the target of excessive oxidative damage that evolves into retinal pathology^14^. Photoreceptor cells express the elongase enzyme ELOVL4 (ELOngation of Very Long chain fatty acids-4), which is evolutionarily conserved in the retina^15^ and catalyzes the biosynthesis of very long chain polyunsaturated fatty acids (≥C28) including n-3 (VLC-PUFAs,n-3) from 26:6 fatty acids derived from DHA or eicosapentaenoic acid (EPA)^16, 17^; EPA has been shown to be the preferred substrate^16^. Even though the levels of EPA are quite low in the retina compared to DHA, retroconversion of DHA to EPA in peroxisomes takes place, and it is possible that EPA produced by this reaction will generate the 26:6 substrate for ELOVL4^16^. These fatty acids become acyl chains of phosphatidylcholines and sphingolipids and are enriched in the inner segment of PRCs. ELOVL4 synthesizes VLC-PUFAs in the retina^18–20^ and testes^21^, and it synthesizes VLC saturated fatty acids (VLC-SFAs) in the skin and brain^22, 23^.

Mutant ELOVL4 causes juvenile macular degeneration in autosomal dominant Stargardt’s disease (STGD3), with loss of central vision, progressive degeneration of the macula and peripheral retina^18–20, 22–28^, and early functional defects in RPE cells and PRCs^29^. Also, recent studies have linked spinocerebellar ataxia to ELOVL4 mutations^30–32^. Moreover, recessive mutations in ELOVL4 result in impaired neural development, neuronal dysfunction, hyper-excitability and seizures^28, 33^, and neuroichthyotic disorders^34^. In addition, ELOVL4 is necessary in the skin-permeability barrier and neonatal survival^23^.

One of the proposed mechanisms for PRC degeneration is that mutations in ELOVL4 that cause dominant Stargardt’s disease are due to the loss of its C-terminal endoplasmic reticulum (ER) retention signal, leading to protein mislocalization and aggregation^18, 19, 28, 35–37^. Thus, mislocalization of the truncated ELOVL4 protein causes cellular stress that leads to PRC death. Alternatively, mislocalization of an enzymatically-active truncated ELOVL4 protein from the ER leads to accumulation of toxic products (*i.e*., 3-keto intermediates) because the truncated protein still contains the putative active site. Production and accumulation of these toxic keto intermediates by the truncated ELOVL4 could be an additive insult to the overall reduction in the ELOVL4-derived products (*i.e*., VLC-PUFAs). Furthermore, ELOVL4 knockout (KO) mice have VLC-PUFA-deficient PRC terminals with reduced rod terminal vesicles and a disorganized outer plexiform layer^38, 39^. The ELOVL4 protein is targeted via its C-terminal di-lysine motif KXKXX to the ER for elongation by a four-step cyclical process of condensation, reduction, dehydration and reduction, yielding a fatty acid elongated by two carbons. The initial condensation reaction and rate-limiting step is catalyzed by an elongase and mediated by iron-coordinating histidines in the active site, which condenses malonyl CoA (the two-carbon donor) and a fatty acyl-CoA to yield a 3-keto-acyl-CoA intermediate. The 3-keto compound is then reduced to the 3-hydroxy product, dehydrated to a trans-2,3-enoyl fatty acyl-CoA, which is further reduced to form the final product, a fatty acid that is two carbons longer than the precursor. The initial and final reduction steps are catalyzed by 3-keto-acyl-CoA reductase (KAR), trans-2,3-enoyl-CoA reductase (TER) enzymes, respectively, both of which require NADPH as a cofactor. The dehydration step is carried out by one of four different 3-hydroxyacyl-CoA dehydratases (HACD1, HACD2, HACD3, or HACD4), and the chain length of the final product is determined by the particular elongase that catalyzes the reaction.

After VLC-PUFAs are generated via ELOVL4, they are incorporated into phospholipids in the PRC inner segment, where they become part of the PRC outer membrane biogenesis^20^ and tightly interact with rhodopsin^40^. Additionally, VLC-PUFAs are assumed to be important in the overall functions of PRC, including longevity, synaptic function, and neuronal connectivity. However, the molecular mechanisms by which VLC-PUFAs exert these important functions and its protective role remain unknown. Herein, we have explored an alternative mechanistic rationale for the significance of ELOVL4 in PRC survival. The genetic ablation of adiponectin receptor 1 (AdipoR1) leads to the depletion of the phosphatidylcholine molecular species (PCMS) that contain 32:6n3, 34:6n3, and DHA (22:6n3), which in turn leads to photoreceptor degeneration that resembles various forms of human retinal degenerative diseases^41^. Thus, a shortage in the protective bioactive mediators derived from VLC-PUFAs may be a fundamental factor in the onset and early progression of these diseases.

## ELV-N32 and ELV-N34 formation, structure and stereochemistry in primary human RPE cells

**Figure 1:**
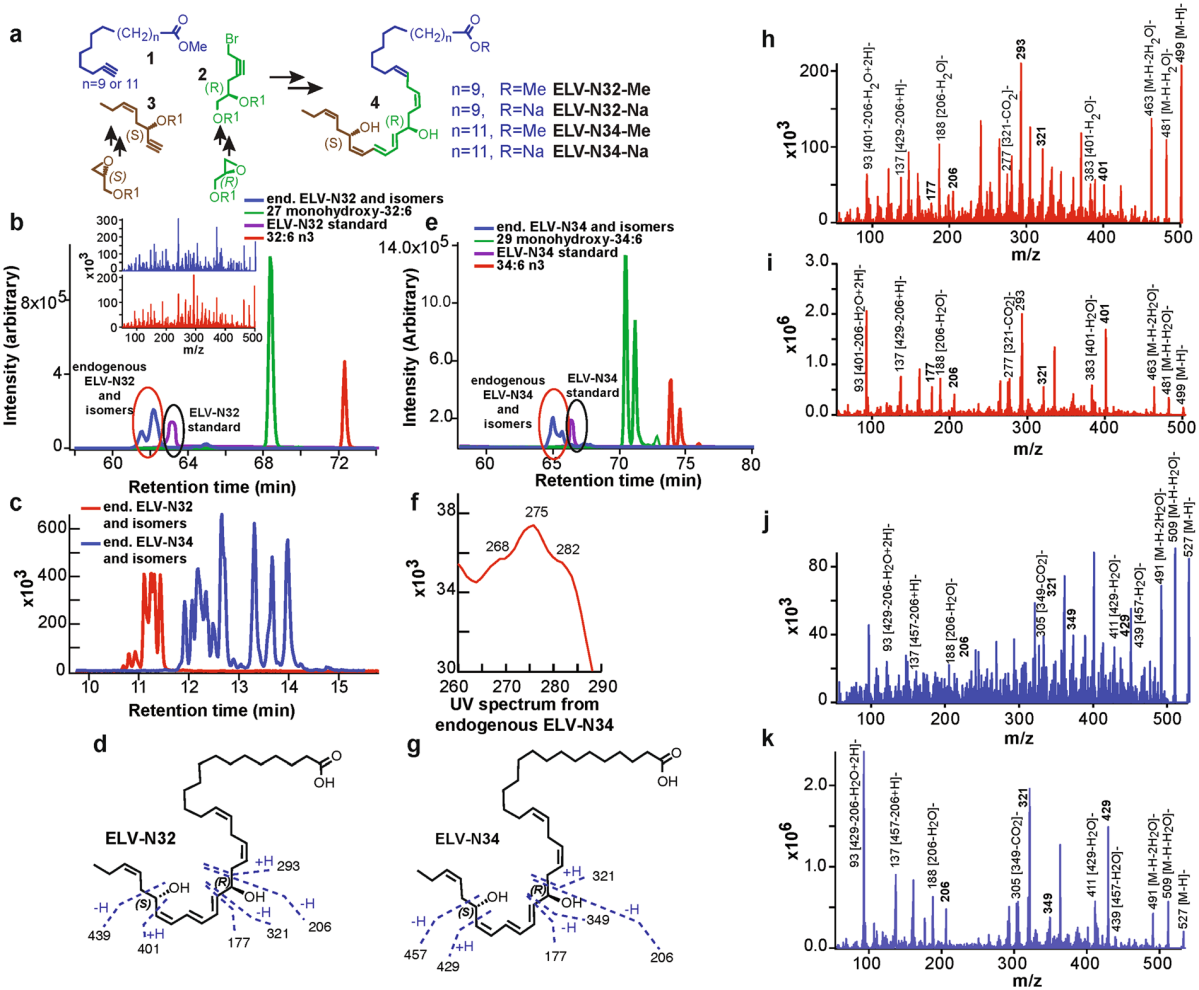
Discovery and structural characterization of ELV-N32 and ELV-N34 in primary human RPE cells in culture. (**a**) ELV-N32 and ELV-N34 were synthesized from three key intermediates (**1**, **2**, and **3**), each of which was prepared in stereochemically-pure form from readily-available starting materials. The stereochemistry of intermediates **2** and **3** was pre-defined by using enantiomerically-pure epoxide starting materials. The final ELVs (**4**) were assembled via iterative couplings of intermediates **1**, **2**, and **3**, and were isolated as methyl esters (Me) or sodium salts (Na). **(b**) 32:6n3 (red line), endogenous mono-hydroxy-32:6n3 (green line), and ELV-N32 (blue line) are shown with the ELV-N32 standard (purple). Multiple reaction monitoring of ELV-N32 shows two large peaks eluted earlier than the peak when standard ELV-N32 was eluted, displaying the same fragmentation patterns (shown in the insert spectra), suggesting that they are isomers. **(c)** Chromatogram for full daughter scans for ELV-N32 (red line) and ELV-N34 (blue line). (**d**) Fragmentation pattern of ELV-N32. **(e)** Same features as in (**b**) for 34:6n3 and ELV-N34. (**f**) UV spectrum of endogenous ELV-N34 showing triene features. **(g**) Fragmentation pattern of ELV-N32. (**h**) Full fragmentation spectra of endogenous ELV-N32, and **(i)** the ELV-N32 standard shows that all major peaks from the standard match to the endogenous peaks. However, endogenous ELV-N32 has more fragments that do not show up in the standard, suggesting that it includes different isomers. (**j**) For ELV-N34, full fragmentation spectra of endogenous ELV-N34 peaks match up with the standard ELV-N34 (**k**), also suggesting the existence of ELV-N34 isomers.

**Figure 2:**
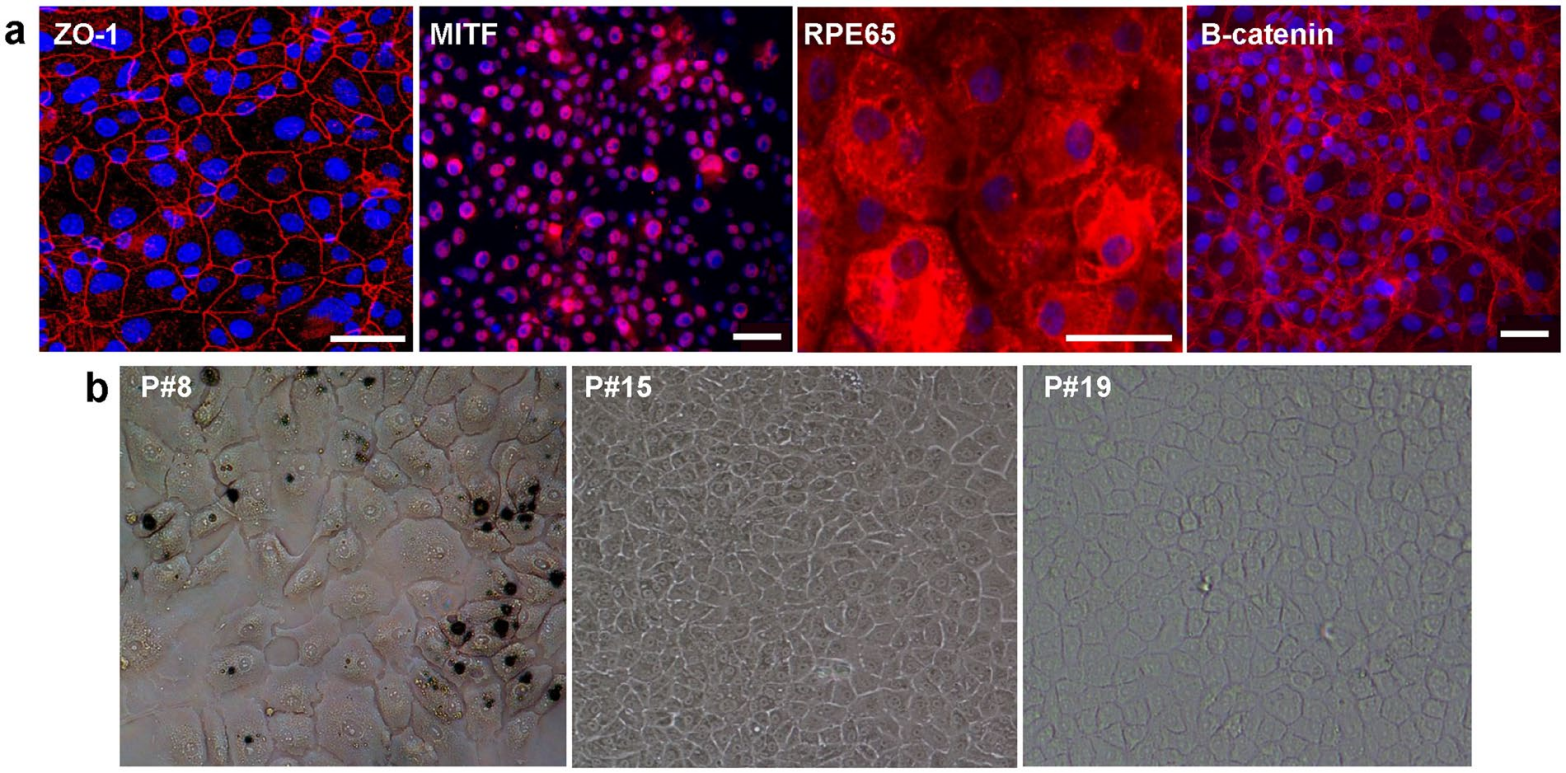
Primary human RPE cells. (**a**) Confocal images of immunostained primary human RPE cells using specific markers ZO-1 (Zona occludens-1), RPE65 (retinal pigment epithelium-specific 65 kDa protein), MITF (Microphtalmia-associated Transcription Factor), and β-catenin. (**b**) Light microscopy depicting primary human RPE cell morphology at different passages in culture. Scale bars, 50 μm.

The complete structures and stereochemistry of the novel 32- and 34-carbon elovanoids ELV-N32 and ELV-N34 were established through a direct comparison with compounds prepared via stereo-controlled total organic synthesis by adapting our previously reported methodologies for the total synthesis of the DHA-derived lipid mediator, neuroprotectin D1 (NPD1; 10*R*,17*S*-dihydroxydocosa- (4*Z*,7*Z*,11*E*,13*E*,15*Z*,19*Z*)-hexaenoic acid)^42, 43^. Further validation of these structural assignments was established by synthesizing deuterium-labelled derivatives (ELV-N32-d2 and ELV-N34-d2) for liquid chromatography tandem mass spectrometry (LC-MS/MS) analysis. ELV-N32 and ELV-N34 were prepared by stereo-controlled total chemical synthesis (Fig. 1a). The availability of synthetic materials with fully-defined structures and stereochemistry allowed us to determine the complete R/S configuration as well as the Z/E geometry of the double bonds in these human primary RPE cell-derived elovanoids (ELVs). Confocal images of immunostained primary human RPE cells (using the specific markers ZO-1 (Zona occludens-1), RPE65 (retinal pigment epithelim-specific 65 kDa protein), MITF (Micro-ophtalmia-associated Transcription Factor) and β-catenin) are depicted in Fig. 2, as well as light microscopy morphology at different passages in culture. In brief, these cells were cultured for 24 to 48 hours followed by a 24-hour incubation with 10 μM free 32:6n6 plus 34:6n6. Then cells were incubated with 1 mM H_2_O_2_ for 24 hours after a 24-hour serum deprivation. The incubation media were collected, and lipids were extracted and loaded onto a liquid chromatography tandem mass spectrometer for analysis. We also generated synthetic stereochemically-pure deuterium-labeled ELVs, and by matching them with endogenously-produced molecules by LC-MS/MS, we further confirmed their structure and stereochemistry. Following matching with human primary RPE cell culture media-derived elovanoids, the complete structures of ELV-N32 (from a 32 carbon omega-3 polyunsaturated fatty acid) and ELV-N34 (from a 34 carbon omega-3 polyunsaturated fatty acid) were confirmed to be as follows: ELV-N32: (14*Z*,17*Z*,20*R*,21*E*,23*E*,25*Z*,27*S*,29*Z*)-20,27-dihydroxydo-triaconta-14,17,21,23, 25,29-hexaenoic acid; ELV-N34: (16*Z*,19*Z*,22*R*,23*E*,25*E*,27*Z*,29*S*,31*Z*)-22,29-dihydroxytetra-triaconta-16,19,23, 25,27,31-hexaenoic acid.

Both of these elovanoids and their precursor VLC-PUFAs were detected in RPE cells under uncompensated oxidative stress (UOS) (Fig. 1b–k). We used m/z 499 → 93 and 499 → 401 MRM transitions for ELV-N32, and m/z 527 → 93 and 527 → 429 transitions for ELV-N34 for detection. For the corresponding precursors, we used m/z 483 → 385 for 27-hydroxy-32:6n3, and m/z 511 → 413 for 29–hydroxyl-34:6n3. For further identification, we performed full fragmentation on ELVs and found good matches to the standards. In summary, here we show the identification and structural characterization of a novel class of oxygenated lipid mediators derived from 32:6n3 or 34:6n3 in primary human RPE cells; we have named these mediators “elovanoids.” The structure and stereochemistry of the novel elovanoids ELV-N32 and ELV-N34, having structures reminiscent of NPD1, were established using synthetic materials produced by stereo-controlled chemical synthesis. We characterized these ELVs in the incubation media from primary human RPE cells exposed to UOS, including deuterium-labeled ELVs for matching experiments. Elovanoids from longer fatty acid chains also are likely to occur.

## 32:6n3 or 34:6n3 elicits potent cytoprotection

**Figure 3:**
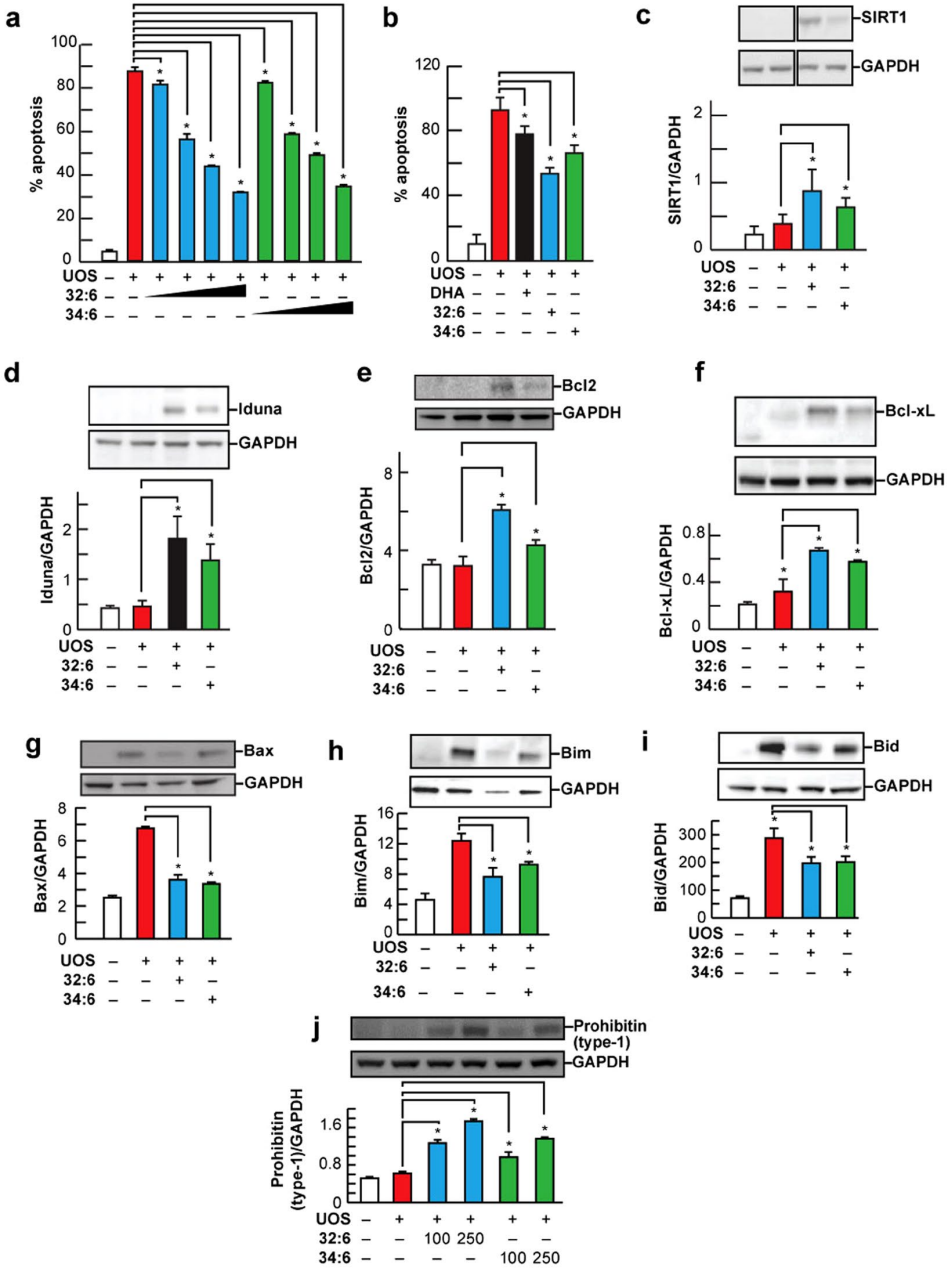
Cytoprotection by 32:6n3 and 34:6n3 in human RPE cells under UOS. (**a**) Concentration-dependent anti-apoptotic activity of 32:6n3 and 34:6n3 in human RPE cells (ARPE-19 cells). Confluent (80%) ARPE-19 cells in 12-well plates were serum starved for 8 hours, UOS was induced and then challenged with 50–500 nM 32:6n3 or 34:6n3 free fatty acids for 16 hours. Treated cells were harvested, and Hoechst-positive pyknotic cells detected as described in the Methods. Data are averages of the counts of 15 wells of Hoechst-positive pyknotic cells of three independent experiments. **(b**) -100nM, DHA-mediated cytoprotection compared with that of 32:6n3 or 34:6n3 (250 nM each) after UOS exposure (16 hours) in serum-starved ARPE-19 cells, as described before. Apoptotic cell death was detected as described above. Results are averages of three independent experiments. **(c)** SIRT1 upregulation by 32:6n3 and 34:6n3 in RPE cells under UOS. The results are the averages of three independent experiments (all together, 9 wells for each experiment) unless otherwise indicated. **(d)** Iduna abundance was enhanced by 32:6n3 or 34:6n3 in RPE cells under UOS. **(e**–**i**) Effect of 32:6n3 or 34:6n3 on pro- and anti-apoptotic proteins: (**e**) Bcl-2, (**f**) Bcl-xL, (**g**) Bax, (**h**) Bim, and (**i**) Bid in ARPE-19 cells under UOS. Western blot detection of the effect of 32:6n3 and 34:6n3 on the up- and down-regulation of the above proteins in ARPE-19 cells under UOS. (**j**) Concentration-dependent (100 and 250 nM) upregulation of prohibitin (type-1) abundance induced by 32:6n3 and 34:6n3 in RPE cells under UOS. Also, see Supplementary Figs 1 and 2.

**Figure 4:**
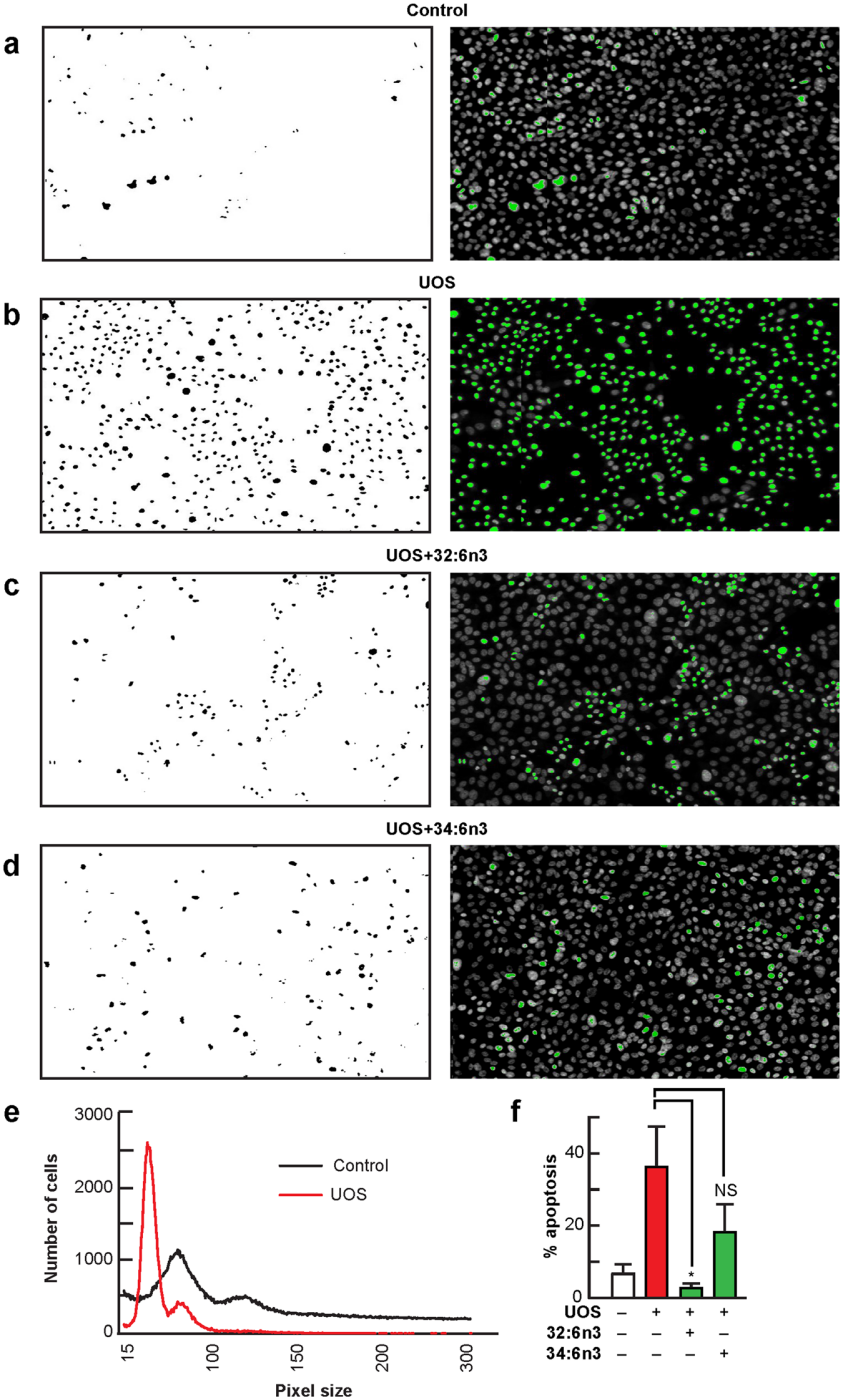
32:6n3 and 34:6n3 ameliorate UOS-induced primary human RPE cell death. (**a**) Untreated (control) RPE cells. RPE cells underwent UOS for 16 hours (**b-f**). When 32:6n3 or 34:6n3 were added, cell death was prevented (**c**,**d**,**f**). Typical fields of cell cultures are represented in the right column. Nuclei are labeled with Hoechst staining, and the dead cells are highlighted in green. These were separated using an intensity threshold algorithm and counted using an Image J macro (left column)^70^. (**e**) Quantification of live (control cells; black curve) and dead (UOS cells, red curve) cells was based on nuclear size^70^. Error bars, SEM; *p < 0.05.

32:6n3 or 34:6n3 are precursors of ELVs, and in fact they are converted into the novel ELVs under our experimental conditions (Fig. 1). Therefore, we asked whether free 32:6n3 or 34:6n3 elicit protection against UOS in RPE cells. To test the efficacy of 32:6n3 and 34:6n3 VLC-PUFAs in modulating human RPE cell homeostasis and survival rates, we incubated human ARPE-19 cells undergoing UOS with both 32:6n3 or 34:6n3 (3 μM each) for 16 hours. The addition of H_2_O_2_ (800 μM) plus tumor necrosis factor alpha (TNFα) (10 ng/ml) induced apoptosis (50% cell death). Both 32:6n3 and 34:6n3 successfully prevented cell death in a concentration-dependent fashion (Fig. 3a). A similar protective effect was observed in primary human RPE cells (Fig. 4).

**Figure 5:**
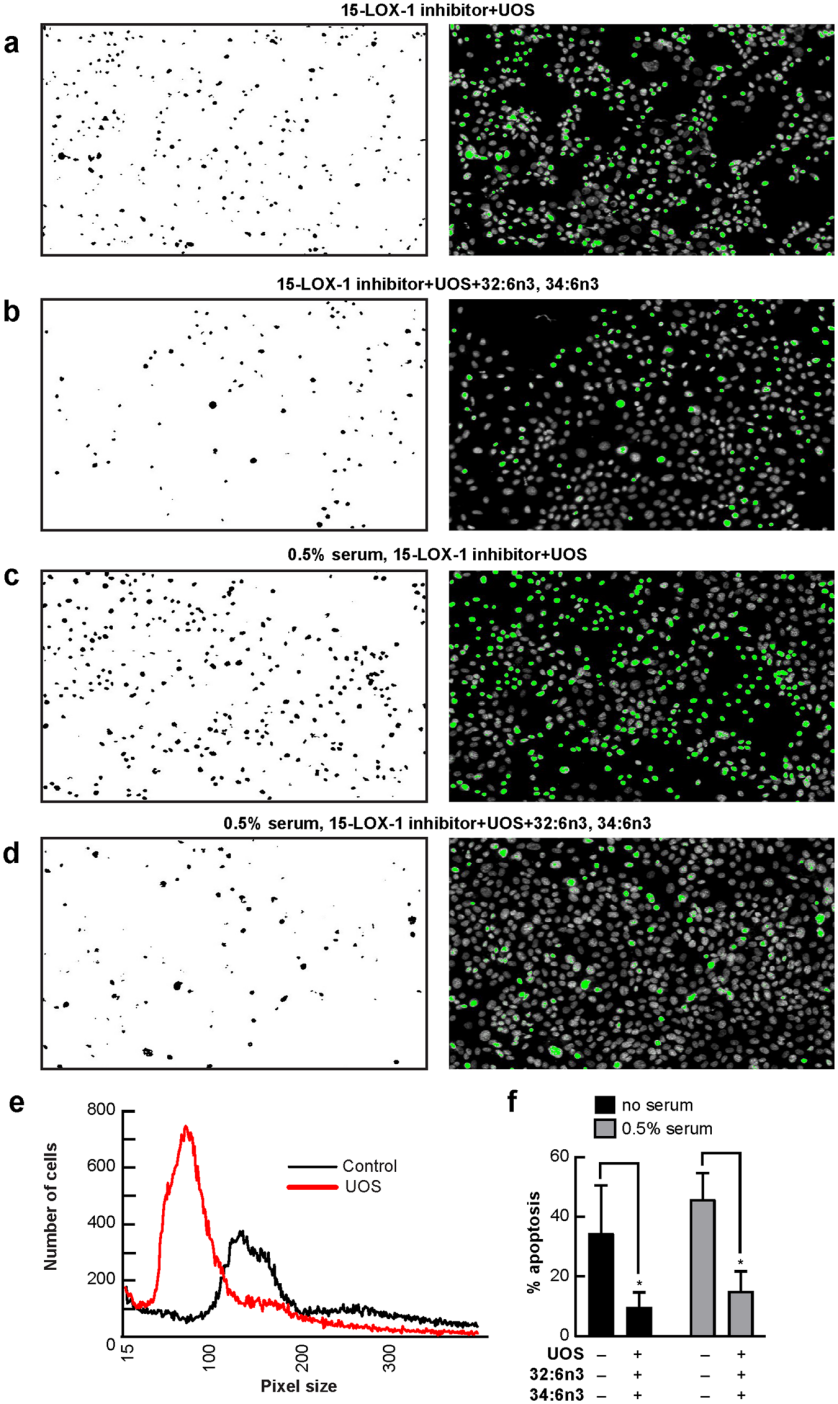
A 15-LOX-1 inhibitor does not modify cytoprotection against UOS mediated by 32:6n3 and 34:6n3 on primary human RPE cells. Serum-deprived (**a**,**b**) and low serum (**c**,**d**) primary human RPE cells were incubated with the 15-lipoxygenase-1 (15-LOX-1) inhibitor (10 micromolar, PD146176) for 1 hour, then subjected to oxidative stress (H_2_O_2_/TNFα) for 16 hours to induce apoptosis (**a**–**d,f**). The addition of 32:6n3 and 34:6n3 protected human RPE cells (**b**,**d**,**f**) from cell death. Typical fields of cell cultures are represented in the right column. Nuclei are labeled with Hoechst staining, and the dead cells are highlighted in green. These were separated using an intensity threshold algorithm and counted using an Image J macro (left column)^70^. (**e**) Quantification of live (control cells; black curve) and dead (UOS cells, red curve) cells was based on nuclear size^70^. Error bars, SEM; *p < 0.05.

Oxidative stress stimulation initiates the enzymatic oxygenation of DHA through the activation of 15-lipoxygenase-1 (15-LOX-1)^44^, leading to the biosynthesis of NPD1^12^. NPD1 is a stress-response lipid mediator derived from DHA^5, 11, 12^, and it enhances survival signaling in RPE cells confronted with oxidative stress by promoting modulation of the activity and content of proteins directly involved in deciding cell fate^9, 10, 44–47^. Primary human RPE cells, formerly serum-deprived for 12 hours, were incubated with the 15-LOX-1 inhibitor (PD146176) (10 μM for 1 hour), then bathed with 600 μM H_2_O_2_/TNFα in conjunction with a mixture of 32:6n3 plus 34:6n3 (3 μM each) for 16 hours (Fig. 5). The 15-LOX-1 inhibitor sensitizes cells; therefore, a lower concentration of H_2_O_2_ than in the cytoprotection experiment was used. As mentioned above, adding H_2_O_2_ and TNFα induced RPE cell apoptosis, but treatment with a mixture of 32:6n3 and 34:6n3 successfully prevented cell death (Fig. 5), indicating that 15-LOX-1 is not involved in this free fatty acid cell protection mechanism using primary human RPE cells; this issue remains to be defined in the future. Thus, newly-identified ELVs are different from other endogenous cytoprotective mediators because, among other reasons, they involve a phospholipid molecular species endowed with acyl chains that are precursors of neuroprotective lipids. DHA, the precursor of the bioactive “docosanoids,” is anchored at position C2 of the glycerol backbone, while 32:6n3 or 34:6n3 are located at position C1 and serve as the reservoir of the precursors of the novel ELVs described herein.

## 32:6n3 and 34:6n3 enhance anti-apoptotic and pro-survival protein expression

**Figure 6:**
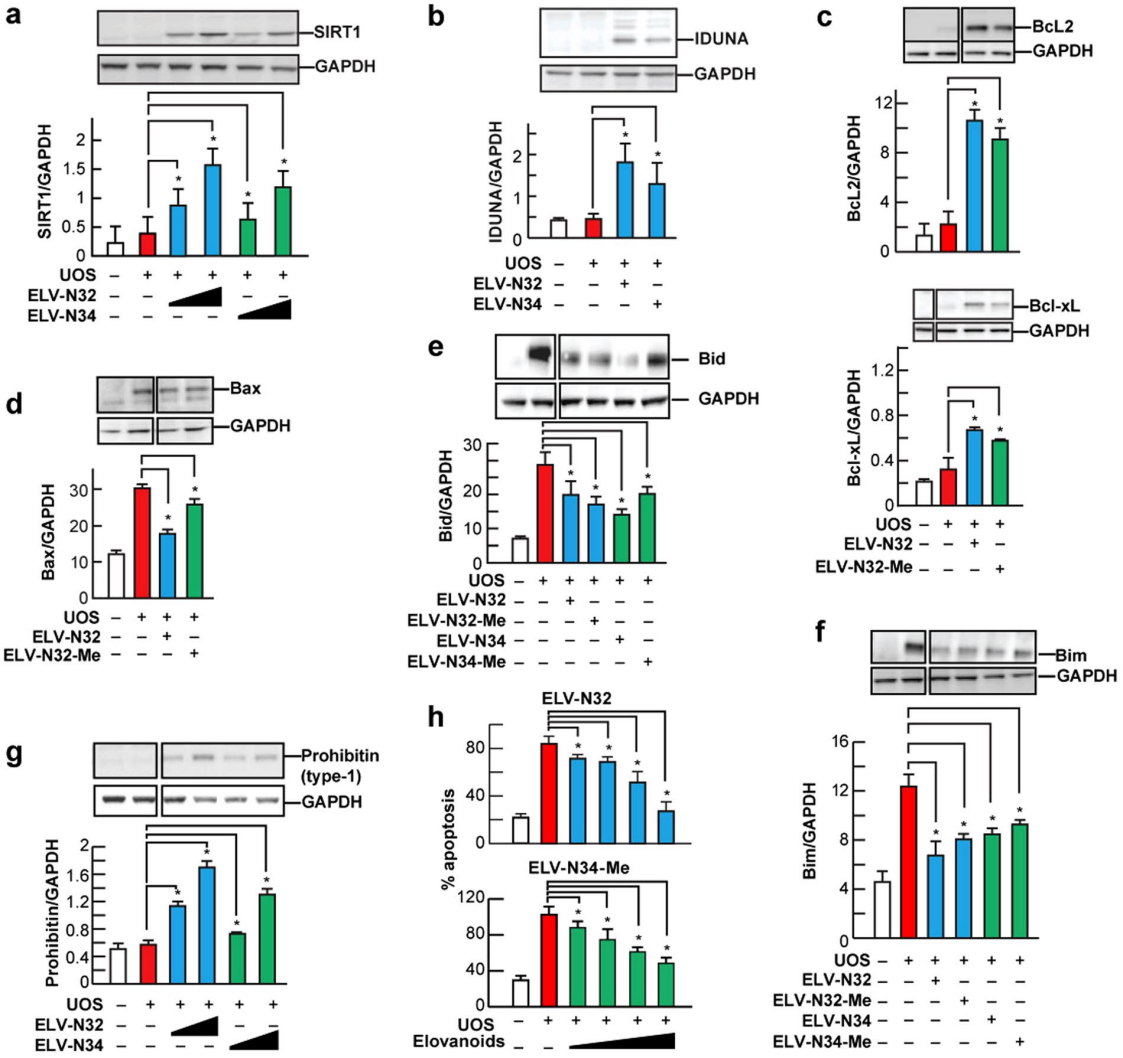
ELV-N32 and ELV-N34 enhance abundance of pro-homeostatic proteins and decrease abundance of cell damaging proteins in RPE cells under UOS. ELV-N32 or ELV-N34 indicates the sodium salt forms, and ELV-N34-Me or ELV-N32-Me indicates the methyl ester forms. ELVs induces the following effects in ARPE-19 cells undergoing UOS: (**a**) Concentration-dependent (100 and 250 nM) upregulation of SIRT1. The results are the averages of three independent experiments; (**b**) upregulation of Iduna abundance; (**c**) increased abundance of anti-apoptotic proteins Bcl-2 and Bcl-xL; (**d**) decreased abundance of pro-apoptotic proteins Bax, (**e**) Bid and (**f**) Bim. (**g**) Concentration-dependent (100 and 250 nM) upregulation of Prohibitin (type-1) by ELVs takes place. (**h**) Concentration-dependent (50, 100, 250, and 500 nM) reduction of UOS-induced apoptosis. Error bars, SEM; *p < 0.05. Also, see Supplementary Figs 4 and 5.

We observed that 32:6n3 and 34:6n3 upregulated the expression of pro-survival Bcl-2 and Bcl-xL (Fig. 3e,f; Supplementary Fig. 1) and down regulated the pro-apoptotic proteins Bax, Bim, and Bid (Fig. 3g–i; Supplementary Fig. 2). Moreover, the pro-homeostatic effects of 32:6n3 and 34:6n3 was concentration-dependent (Fig. 3j; Supplementary Fig. 2), and sirtuin-1 (SIRT1) and Iduna abundance were augmented (Fig. 3c,d; Supplementary Fig. 1). So since 32:6n3 or 34:6n3 are the precursors of ELV-N32 and ELV-N34, respectively, under the present experimental conditions we interpreted this to mean that these results are mediated by ELVs (Fig. 1). Moreover, in Fig. 6 (Supplementary Figs 3 and 4), we tested similar targets using ELVs and found similar results. Since sirtuins have been shown to be involved in retinal disease^48^ and play a role in aging^49, 50^, mitochondrial function^51^, and overall homeostasis^52^, this protein is important in eliciting the biological activities of the novel lipid mediators. The other protein targeted is Iduna (ring finger protein 146 (RNF146). Iduna is a PARsylation-directed ring finger E3 ubiquitin ligase engaged in protein quality control and DNA repair, and it facilitates protection against parthanatos^53, 54^, which is a form of cell death dependent on poly(ADP-ribose) polymerase-1 (PARP-1)^53, 55–57^. PARPs catalyze the transfer of ADP-ribose from nicotinamide adenine dinucleotide (NAD) to target proteins and are indispensable for genomic integrity, the cell cycle, and gene expression^58^. Recently, it was found that NPD1 augments the abundance of Iduna in RPE cells when confronted with UOS^59^.

## ELVs upregulate pro-homeostatic and anti-apoptotic protein abundance with attenuation of apoptosis in RPE

VLC-PUFAs play a vital role in PRC structure and function, which are compromised when DHA levels are reduced due to genetic ablation of AdipoR1^41^. A consequence of this is that retinal degeneration ensues. To counter neurodegeneration, cells activate neuroprotective pathways that sustain a balance between pro- and anti-apoptotic signaling. The genetic and cellular mechanisms that govern the expression of these pro-survival mechanisms, in addition to the mediators that signal to proteins that carry protective actions, are not known. Therefore, we next explored whether ELVs enhance the expression of pro-survival and pro-homeostatic proteins in RPE cells undergoing UOS. Figure 6 shows that either the sodium salt or the methyl ester form of ELVs potently regulate the abundance of several key proteins. ELV-N32-Na and ELV-N34-Na upregulated SIRT1 abundance in UOS RPE cells in a dose-dependent manner (100–200 nM) (Fig. 6a; Supplementary Fig. 3), and they also enhanced Iduna expression in RPE cells under UOS at concentrations of 200 nM (Fig. 6b; Supplementary Fig. 3). ELV-N32-Na or ELV-N34-Na also enhanced the abundance of the anti-apoptotic proteins Bcl-2 and Bcl-xL (Fig. 6c; Supplementary Fig. 3). On the other hand, pro-apoptotic Bax (Fig. 6d; Supplementary Fig. 4), Bid (Fig. 6e; Supplementary Fig. 4), and Bim (Fig. 6f; Supplementary Fig. 4) were decreased by ELV-N32 or ELV-N34 (with the sodium salts or methyl esters). It is interesting to note that while Bcl-2 and Bcl-xL were upregulated (Fig. 6c; Supplementary Fig. 3), Bax, Bim, and Bid were downregulated by either the sodium salts or methyl esters (Fig. 6d–f; Supplementary Fig. 4). Prohibitin (type-1), a cell-survival protein^47, 60–62^, was upregulated by both ELV-N32 and ELV-N34 (sodium salts and methyl ester forms) in a concentration-dependent manner (100–200 nM) in RPE cells undergoing UOS (Fig. 6g; Supplementary Fig. 4). We have shown here that ELV-N32 or ELV-N34 (sodium salts or methyl esters) upregulate SIRT1 and Iduna proteins, while inhibition of apoptosis takes place in RPE under UOS. These observations suggest that ELVs may be playing an important role in cell signaling^5, 13^. Moreover, the 32:6n3 and 34:6n3 cytoprotective response was not affected by the presence of the 15-LOX-1 inhibitor in the primary human RPE cells. ELV upregulation of the expression of Bcl-2 and Bcl-xL, and ELV downregulation of Bax, Bim and Bid in RPE cells undergoing UOS, indicates that ELVs are involved in modulating cell apoptotic pathways^18, 23^. Moreover, Fig. 6h shows that ELV-N32-Na and ELV-N34-Me attenuate apoptosis in RPE cells in a concentration-dependent fashion (50–500 nM). The highest inhibition was at 500 nM (for both the sodium salt and methyl ester forms) and the lowest was at 50 nM (Fig. 6h). Furthermore, the increased SIRT1 abundance induced by 32:6n3 and 34:6n3 (Fig. 3c; Supplementary Fig. 1) and by ELVs (Fig. 6a; Supplementary Fig. 3) highlights an additional target of these novel mediators on pro-homeostatic bioactivity. The observation that ELVs upregulated prohibitin (type-1) in these RPE cells undergoing UOS is of interest in the biology of senescence and cell survival. Prohibitins are ubiquitous, evolutionarily-conserved proteins that form a ring-like, high-molecular-mass complex at the inner membrane of mitochondria and other cellular compartments^60–63^. In addition, they also are involved in energy metabolism, proliferation, apoptosis, and senescence^30^. Prohibitin regulates signaling of membrane transport, control of transcription activation, and the cell cycle, while the mitochondrial prohibitin complex stabilizes the mitochondrial genome and modulates mitochondrial dynamics, morphology, biogenesis, and the intrinsic apoptotic pathway^63^. Therefore, we suggest that by manipulating the intracellular abundance of prohibitin (type-1), ELVs could provide a possible way to control aging and other pro-homeostatic functions in mammalian cells.

## AdipoR1 regulates DHA uptake and ELV formation

**Figure 7:**
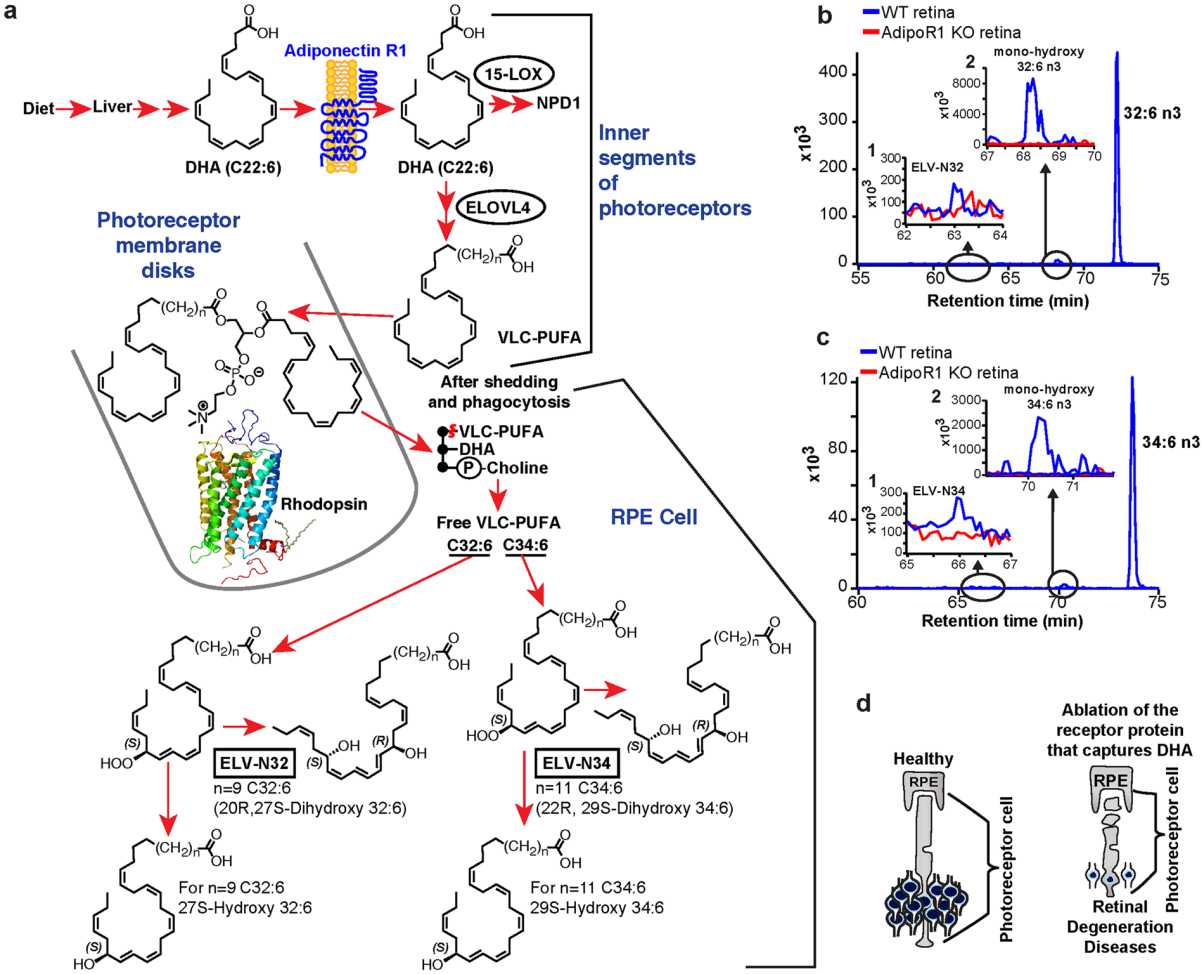
Genetic ablation of adiponectin receptor 1 leads to depletion of VLC-PUFAs and its derivatives in retina. (**a**) Dietary DHA, or that derived from dietary 18:3n3, is supplied by the liver and captured by adiponectin receptor 1 (AdipoR1), followed by elongation in the inner segment of PRC by ELOVL4 to VLC-PUFA and incorporation into phosphatidylcholine molecular species, which also contains DHA. During daily PRC outer segment renewal, these phosphatidylcholine molecular species interact with rhodopsin and, after shedding and phagocytosis, become part of RPE cells. UOS or other disruptors of homeostasis trigger the release of VLC-PUFAs. 32:6n3 and 34:6n3 are depicted generating hydroperoxy forms, and then ELV-N32 or ELV-N34, respectively. (**b)** The pool size of free 32:6n3 in retinas of AdipoR1 knockout (KO) mice (red) is decreased as compared with that in wild type (WT) (blue). Insert (**1**) shows ELV-N32 in KO (red) and WT (blue); insert (**2**) shows mono-hydroxy 32:6n3, the stable derivative of the hydroperoxy precursor of ELV-N32, in WT (blue) and lack of detectable signal in the KO (red). **(c)** Similarly, the pool size of free 34:6n3 in retinas of AdipoR1 KO mice (red) is decreased as compared with that in WT (blue). Insert (**1**) shows ELV-N32 in KO (red) and WT (blue); insert (**2**) shows mono-hydroxy 34:6n3, the stable derivative of the hydroperoxy precursor of ELV-N34, in WT (blue) and lack of detectable signal in the KO (red). **(d**) RPE cells sustain PRC functional integrity (left); right, the ablation of AdipoR1 switches off DHA availability, and PRC degeneration ensues.

RPE cells sustain PRC functional integrity, and their demise is involved in the onset of several forms of retinal degenerations (Fig. 7d). One of the functions of the RPE cell is to retrieve DHA during PRC renewal and return it through the interphotoreceptor matrix to the PRC inner segment for new outer segment disc membrane biogenesis^64^. Recently, adiponectin receptor 1 (AdipoR1) was found to be necessary for DHA availability to photoreceptor cells^41^, and a single amino acid mutation in this receptor is causative of autosomal dominant retinitis pigmentosa^65^. Genetic ablation of this receptor leads to PRC degeneration and to shutting off VLC-PUFA,n-3 synthesis in the retina. Here we show in Fig. 7 that the pool size of free 32:6n3 and of 34:6n3 in retinas of AdipoR1 knockout (KO) mice (red) is drastically decreased as compared with that in WT (blue). Moreover, ELV-N32 and ELV-N34 in KO (red) were undetectable. Mono-hydroxy 32:6n3 and 34:6n3, the stable derivatives of the hydroperoxy precursors of ELV-N32 and of ELV-N34 respectively, lack a detectable signal in the KO (red), unlike the wild type (blue) (Fig. 7b,c). ELV-N32 and ELV-N34 were found to be secreted from RPE cells when confronted with UOS, suggesting paracrine or autocrine bioactivity. We also show that these ELVs target and enhance the expression of pro-survival and pro-homeostatic proteins in the RPE cells undergoing UOS.

## ELVs protect RPE cells, which sustain PRC integrity

PUFA elongation in the inner segment of photoreceptors by ELOVL4 leads to the biosynthesis of VLC-PUFAs,n-3 and their insertion at the C1 position of phosphatidylcholine within PRC disk membranes. However, under conditions of stress, these VLC-PUFAs are cleaved by phospholipase A1 (PLA1) for the synthesis of mono- and di-hydroxy VLC-PUFAs (ELVs) (Fig. 7a). Light-induced oxidative stress in mouse retinas triggers the production of free 32:6n3 and 34:6n3, as well as their mono- and di-hydroxy derivatives (Fig. 7a). In AdipoR1 KO mice, no detectable amounts of these molecules were found (Fig. 7b,c, red curves). Therefore, the lack of the VLC-PUFA,n-3 precursor DHA results in retinal degeneration (Fig. 7d)^41^, preceded by a remarkable downregulation of the free VLC-PUFA,n-3 molecular species and ELV biosynthesis. These observations support our present hypothesis that VLC-PUFA,n-3 are precursors of novel bioactive mediators that elicit pro-homeostatic protective bioactivity.

## Concluding Remarks

Here we report the discovery of elovanoids (ELVs), the first bioactive lipid mediators derived from VLC-PUFA,n-3, which are the biosynthetic products of elongase ELOVL4. We established the structure and stereochemistry of ELVs with 32 and 34 carbons (ELV-N32, ELV-N34) using synthetic materials obtained by stereocontrolled total synthesis. ELV availability is abolished in the retinas of mice with genetically-ablated AdipoR1 (Fig. 7). Dietary DHA (or derived from dietary 18:3n3) is supplied to tissues by the liver and captured by AdipoR1, followed by elongation in the inner segment of photoreceptor cells by ELOVL4 to VLC-PUFA,n-3 and incorporation into phosphatidylcholine molecular species, which are also endowed with DHA. ELOVL4 uses EPA as a preferred substrate^16^, in spite of the fact that the abundance of EPA is very low in the retina compared to DHA. Retroconversion of DHA to EPA in peroxisomes has been demonstrated, and EPA generated by this reaction might generate the 26 carbon PUFA that is the substrate for ELOVL4^16^. During daily PRC outer segment renewal, these phosphatidylcholine molecular species interact with rhodopsin^40^ and, after shedding and phagocytosis, become part of the RPE cells. UOS or other disruptors of homeostasis triggers the release of VLC-PUFAs. Figure 7a shows that 32:6n3 and 34:6n3 generate a hydroperoxy molecule and then an ELV-N32 or ELV-N34, respectively.

ELVs are biosynthesized in human RPE cells and have protective functions in RPE cells undergoing UOS (Figs 4, 5 and 7). The bioactivity targets of ELVs in RPE cells reveal strong pro-homeostatic functions. The discovery of ELVs points to previously unknown pathways for preserving PRC integrity. ELV formation likely involves an alternative activation pathway since VLC-PUFA,n-3 are incorporated at position C1 of phospholipids, while DHA is located at position C2. ELV biosynthesis, in combination with alternative regulation by PRC-specific phospholipases A1 and A2, point to a novel neuroprotective mechanism in the retina. We did not detect VLC-PUFAs n-3 in sphingomyelin or in other phospholipids, such as phosphatidylethanolamine and phosphatidylserine.

Our data show that the availability of free VLC-PUFA n-3 plus UOS leads to the activation of pro-homeostatic events and RPE cytoprotection. We characterized the synthesis of the novel lipids biosynthesized under these conditions, including the S-precursor and the stable analogs of hydroxyl-derivatives^66^. Many new questions emerge from our observations: Are phospholipases A1 and A2 modulated in a coordinated fashion? Are neurotrophins a modulator of the pathways that these enzymes create, as in the case of NPD1 synthesis^47, 67^? If so, how are neurotrophins instructed to cleave C1 or C2? Are there synergies between NPD1 (or other docosanoids) and elovanoids? The novel ELV bioactive lipids disclosed here (ELV-N32 and ELV-N34) involve the prior release of either 32:6n3 or 34:6n3 from the C1 position of the phosphatidyl choline. Since this phospholipid molecular species also has DHA in the C2 position, we suggest that NPD1 also can be made from the same precursor. Therefore, we reveal here a different signal bifurcation mechanism that aims to sustain PRC and RPE cell integrity. This is supported by our observation that genetic ablation of AdipoR1, which results in depletion of molecular species of PRC that contains 32:6n3 or 34:6n3 and DHA in the mouse, leads to photoreceptor degeneration resembling various human forms of retinal degenerative diseases^41^. We anticipate that other ELVs also might be made to regulate cell function in other cells.

Mutant ELOVL4 causes juvenile macular degeneration and other neurological conditions. Among the proposed mechanisms for photoreceptor cell degeneration caused by mutant ELOVL4 is the loss of its C-terminal ER retention signal, leading to protein mislocalization of the truncated ELOVL4 protein that, in turn, causes cellular stress that leads to photoreceptor cell death. The data presented here suggest an alternative mechanism for the deleterious effects of mutant ELOVL4, which would limit the occurrence of VLC-PUFA,n-3 in the C1 position of phosphatidylcholines and sphingolipids. Thus, VLC-PUFAs,n-3 are converted to the corresponding ELVs, which are protective in cell survival under UOS conditions. The RPE and retina, under continuous stress, might need ELVs to sustain the functional integrity of RPE cells and the overall function of photoreceptor cells: vision.

The bioactivities of ELV-N32 and ELV-N34 include some unusual and unique features. In addition to their potent neuroprotective actions, these lipid mediators: (a) are cell selective; (b) involve a relationship between PRC and RPE cells that is necessary for vision; (c) are derived from VLC-PUFA,n-3, the biosynthesis of which is regulated by a PRC-specific enzyme, ELOVL4; and (d) have precursor fatty acids (VLC-PUFA,n-3) that are positioned as acyl chains at position C1 of the phosphatidylcholine, unlike DHA (the precursor of NPD1), which is incorporated at position C2. Since they are derived from an alternative fatty acid precursor regulated by ELOVL4 and stored at an alternative phospholipid position, the ELVs are likely to involve an alternative activation pathway for exerting their neuroprotective bioactivity in the retina.

Another significant question raised by our novel findings is as follows: Which signaling mechanism targets the novel phosphatidylcholine molecular species that, after shedding and phagocytosis, appears in RPE cells? The phosphatidylcholine molecular species in the RPE cell stores precursors of two lipid mediators, DHA in the C2 position, and VLC-PUFAs 32:6n3 or 34:6n3 (the precursors of ELV-N32 and ELV-N34) in the C1 position. The phosphatidylcholine molecular species is targeted for release of the acyl chains at C1 and C2 when confronted with UOS as in the onset of retinal degenerations. The new ELVs reported here provide a novel autocrine/paracrine pro-homeostatic RPE signaling that aims to sustain PRC and RPE cell integrity, thus revealing the potential for developing novel therapeutic approaches for retinal degenerations.

## Methods

### Experimental approval

All animal experiments conducted were approved by the Institutional Animal Care and Use Committee of Louisiana State University Health New Orleans (LSUHNO), and all experiments involving primary human retinal pigment epithelia (RPE) cells were approved by the Institutional Review Board of LSUHNO; all experiments were conducted in accordance with National Institutes of Health guidelines. Cells were collected from anonymous human donors provided by eye banks, thus the identity of the donors was unknown.

### Antibodies

The following antibodies were used: β-catenin (catalog# sc-7963, lot# K0812) Santa Cruz Biotechnology: (concentration used 1:50); ZO-1 (catalog# 187430, lot# 1633993 A) Life Technologies: (concentration used 1:100); MITF (catalog# ab59232, lot# GR52475-3) ABCAM: (concentration used 1:250); RPE65 (catalog# ab78036, lot 3GR254004-1), ABCAM: (concentration used 1:250).

### Human RPE cell cultures

Globes of a 19-year-old Caucasian male without eye pathology were obtained from NDRI within 24 hours after death (head trauma). Globes were opened, and then RPE cells were harvested and cultured^68, 69^. Cells from passage 4 were placed in medium containing 10% DMSO and frozen in liquid N_2_. When needed, cells were unfrozen, placed in T75 flasks, and used after passage 8.

Cells were cultured in T75 flasks in MEM medium containing 10% FBS, 5% NCS, MEM-NEAA (ThermoFisher Scientific, Waltham, MA), 1x Penicillin/Streptomycin and 10 ng/ml FGF at 37 °C 5% CO_2_, 99% relative humidityfor 24–48 hours followed by a 24-hour incubation with 10 μM free 32:6n3 and 34:6n3 fatty acid mixture. Figure 2 depicts immunostaining of primary human RPE cells using specific markers ZO-1 (Zona occludens-1), RPE65, MITF (Micro-ophtalmia-associated Transcription Factor) and β-catenin, as well as light microscopy depicting primary human RPE cell morphology at different passages in culture. ARPE-19 cells were grown and maintained in T75 flasks in DMEM F12 medium containing 10% FBS and incubated at 37 °C with a constant supply of 5% CO_2_. Cells at 75–80% confluence (72 hours growth in DMEM/F12 + 10% FBS) in 6-well plates were serum-starved for 8 hours before exposure.

### Exposure of RPE cells to UOS and 32:6n3, 34:6n3 or ELVs

ARPE-19 cells at 75–80% confluence (72 hours growth in DMEM/F12 + 10% FBS) in 6-well plates were serum-starved for 8 hours. Then cells were treated with TNFα (Sigma–Aldrich, St. Louis, MO) (10 ng/ml) and H_2_O_2_ (600 μM) to induce uncompensated oxidative stress either for 6 hours (for Western-blot analysis of selected proteins) or 16 hours (for apoptosis assessment) while the cells were treated with increasing concentrations (50–500 nM) of 32:6n3 and 34:6n3. These fatty acids were applied as follows: the stocks of the free fatty acid forms of 32:6n3, 34:6n3 or ELVs (sodium salt or methyl ester) were dried under N_2_ and resuspended in ethanol. No precipitates were formed. In order to add them to the cell cultures, fatty acids or ELVs were dissolved in medium containing 0.5% serum and incubated with cells. All control samples received appropriate amounts of ethanol; no cell toxicity was observed.

For primary human RPE cell viability assay experiments, cells were treated with 32:6n3 plus 34:6n3 (3 μM each) or separately for the entire duration of the experiment (Figs 5 and 6). For the inhibition studies, the 15-LOX-1 inhibitor (PD146176) (10 μm) was added to the cells 1 hour before oxidative stress induction and kept throughout the incubation period.

### Analysis of proteins

Bcl-2 family proteins, SIRT1 and Prohibitin (type-1), and Iduna proteins were analyze by Western blot analysis. In brief, 20–25 μg equivalents of each cell extracts were subjected to electrophoresis on a 4–12% gels (Promega) at 125 V for 2 hours. The proteins were transferred to a nitrocellulose membrane by an I-blot transfer apparatus. The membranes were subjected to treatment with primary antibodies of Bcl-2, Bcl-xL, Bax, Bid, Bim, SIRT1 and prohibitin (type-1) (Santa Cruz Biotechnology) and Iduna (Neuro-Mab Lab, UCLA, Los Angeles, CA) overnight at 4 °C and probed for 45 minutes with secondary antibody, goat anti-mouse Ig:horseradish peroxidase, and horseradish peroxidase-conjugated anti-biotin antibody, and then proteins were evaluated by using an ECL kit (Amersham).

### Immunocytochemistry and cell apoptosis assessment

Immunocytochemistry assays were performed in 8-well slide chambers. Briefly, cells were fixed in 4% paraformaldehyde (PFA) for 20 minutes, permeabilized with Triton X-100 0.1% in PBS, and non-specific epitopes were blocked in 10% bovine serum albumin (BSA) in 1 × PBS for 1 hour at room temperature. Immunostaining was accomplished by incubating primary antibodies overnight at 4 °C. Samples were incubated for 2 hours at room temperature with Alexa Fluor 555 conjugated secondary antibodies diluted at 1 in 250 (MeridianLife Science Inc., Memphis, TN), and nuclei were stained with Hoechst (2 μM Hoechst33258). Pictures were taken with a Zeiss LSM 510 confocal microscope and a Zeiss Axioplan-2 deconvolution microscope.

To assess cell death, primary human RPE cells and ARPE-19 cells were fixed with methanol for 15 minutes, washed with 1 × PBS, then loaded with 2 μM Hoechst dissolved in a Locke’s solution (Promega) and incubated for another 15 minutes before imaging. Cells were then viewed by using a Zeiss LSM 510 confocal microscope under UV fluorescence. Images were recorded, and cell apoptosis was assessed by using an automated unbiased method^70^.

### LC-MS/MS of ELV-N32 and ELV-N34 in RPE cells

Human RPE cells (at passage 19) were cultured in T75 flasks for 24–48 hours followed by a 24-hour incubation with 10 μM free 32:6n3 and 34:6n3 fatty acid mixture. Cells were incubated with 1 mM H_2_O_2_ for 24 hours promptly after a 24-hour serum deprivation. Fatty acids were extracted using a liquid-liquid lipid extraction method from the collected cell culture medium. Extracts were loaded onto a liquid chromatography tandem mass spectrometer for analysis. We analyzed fatty acids, monohydroxy fatty acid derivatives (27-hydroxy-fatty acid 32:6n3 and 29–hydroxyl-fatty acid 34:6n3), ELV-N32 (20,27-dihydroxy-fatty acid 32:6n3), and ELV-N34 (22,29-dihydroxy-fatty acid 34:6n3). ELV-N32 and ELV-N34 and their deuterium-labeled derivatives, ELV-N32-d2 and ELV-N34-d2, were prepared by stereo-controlled chemical synthesis and used for matching with cell-generated derivatives.

### Photo-oxidative stress

C57BL/6 wild type and AdipoR1 knockout mice were housed in a temperature-controlled room at 21–23 °C with a 12-hour:12-hour light-dark cycle. For light-induced oxidative stress, mice were exposed for 1 hour to bright light (using an 8-light array of 10-inch circular fluorescent 22 W bulbs; Cool White, FTC8T9/CW; General Electric, Fairfield, CT; 18 klux; 270 µE m-2 s). After light exposure, animals were sacrificed by cervical dislocation, and eyes were enucleated. The cornea, iris and lens were discarded and the retina was separated from the rest of the eyecup. These tissues were then flash-frozen. Retinas from animals of the same genotype were pooled together. Samples were processed for lipid extraction and LC-MS/MS-based lipidomic analysis.

## Electronic supplementary material

Supplementary Information
