# Supplementary material for: Elovanoids are novel cell-specific lipid mediators necessary for neuroprotective signaling for photoreceptor cell integrity
Source: Sci Rep. 2017 Jul 13;7:5279. doi: 10.1038/s41598-017-05433-7 (PMC5509689; doi:10.1038/s41598-017-05433-7)
Supplement: Supplementary file 1 — Supplementary Information [file 41598_2017_5433_MOESM1_ESM.docx]

**Supplementary Material**

**Elovanoids are novel cell-specific lipid mediators necessary for neuroprotective signaling for photoreceptor cell integrity**

Bokkyoo Jun^1^, Pranab K. Mukherjee^1^, Aram Asatryan^1^, Marie-Audrey Kautzmann^1^, Jessica Heap^1^, William C. Gordon^1^, Surjyadipta Bhattacharjee^1^, Rong Yang^2^, Nicos A. Petasis^2^ and Nicolas G. Bazan^1^

^1^Neuroscience Center of Excellence, School of Medicine, Louisiana State University Health New Orleans, New Orleans, LA

^2^Department of Chemistry and Loker Hydrocarbon Research Institute, University of Southern California, Los Angeles, CA

**Supplementary Figure 1.** Full length blots of cropped images shown in Figure 3.

**Supplementary Figure 2.** Full length blots of cropped images shown in Figure 3.

**Supplementary Figure 3.** Full length blots of cropped images shown in Figure 6.

**Supplementary Figure 4.** Full length blots of cropped images shown in Figure 6.

**
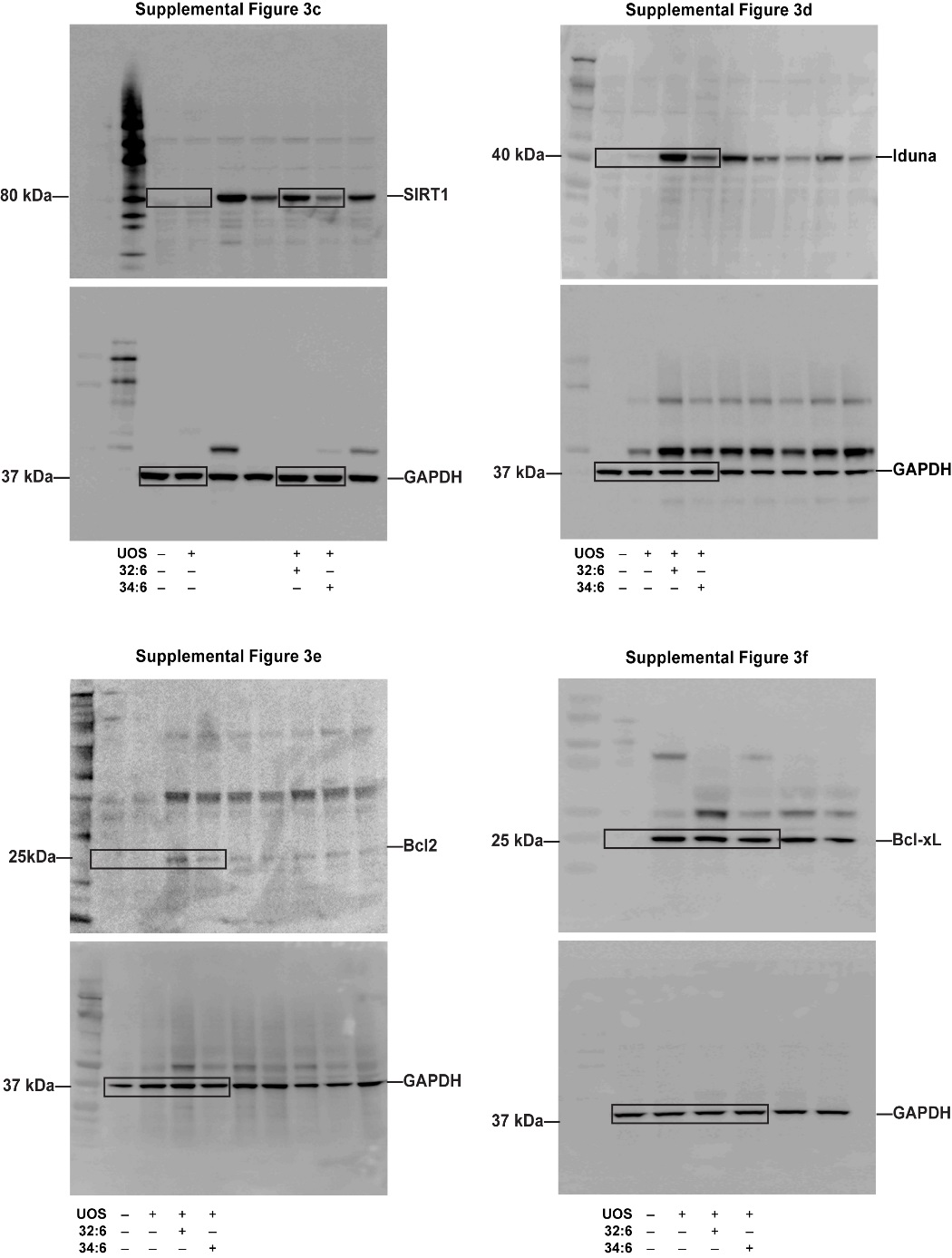
**

**Supplementary Figure 1.** Full length western blots of cropped images shown in Figure 3c, d, e, & f. Western blot analysis of signaling responses in the presence or absence of long chain free fatty acids (LCFAs) C 32:6n3 and C 34:6n3 in retinal pigment epithelial cell lysates using indicated antibodies under uncompensated oxidative-stress (UOS). In brief, 20-25µg equivalents of each cell lysates were subjected to electrophoresis on 4-12% gels (Promega) at 125 volts for 2 hours. The proteins were transferred to nitrocellulose membranes by an I-blot transfer apparatus. The membranes were subjected to treatment with primary antibodies SIRT1 (c), Bcl2 (e), Bcl-xL (f) from Santa Cruz Biotechnology and Iduna (d) from Neuro-Mab Lab, Los Angeles, CA, overnight at 40°C and probed for 45 minutes with secondary antibodies as indicated in Materials and Methods. Subsequently, all blots were stripped and re-probed with anti-GAPDH (Santa Cruz Biotechnology) as a loading control. The bands of interest are indicated by black boxes on the gels. The position of the bands for the respective protein molecular weight markers are indicated to the left of the blots.

**
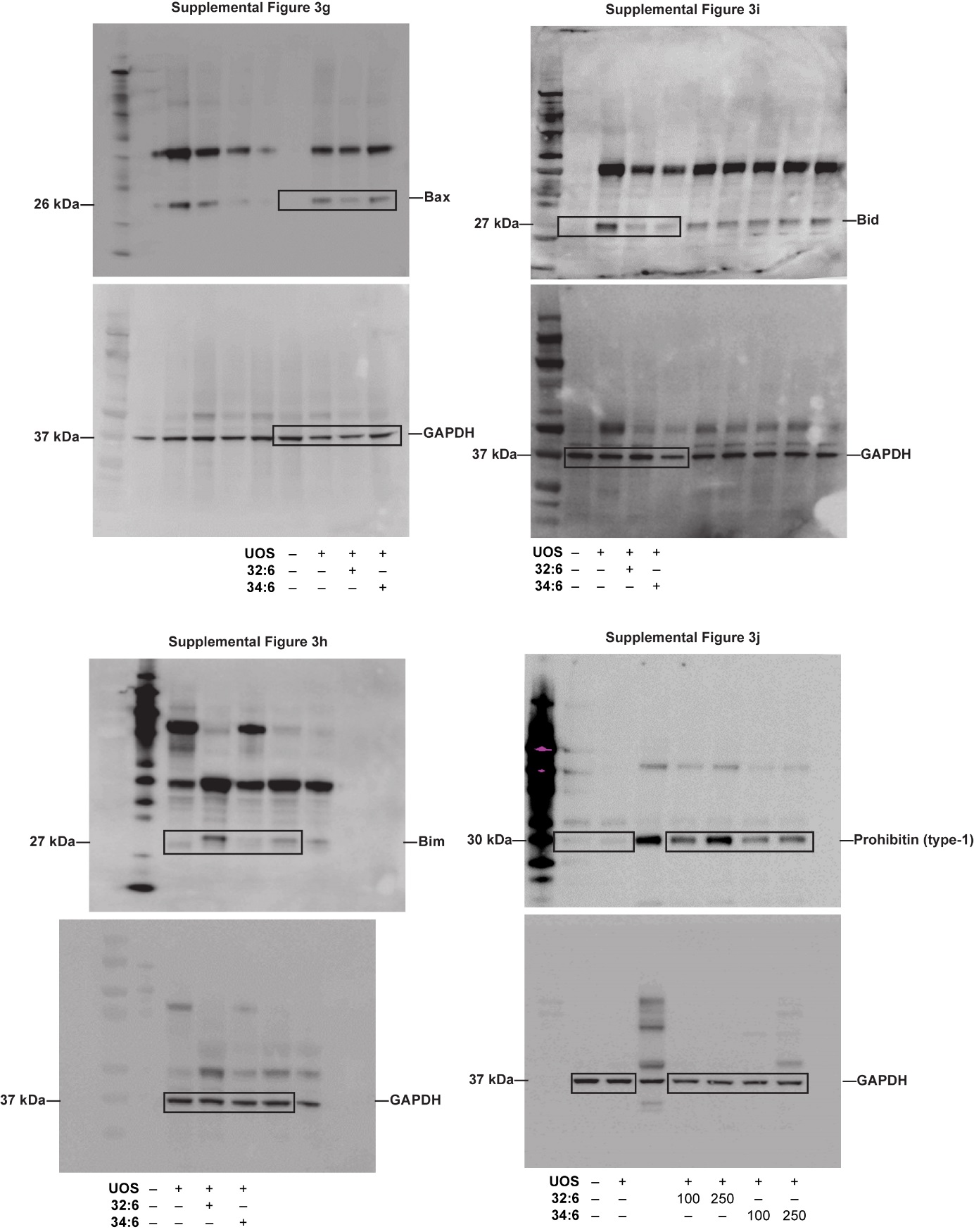
**

**Supplementary Figure 2.** Full length western blots of cropped images shown in Figure 3g, h, i, & j. Methods were the same as described in Supplementary Figure 1. Bax (g), Bim (h), Bid (i), and Prohibitin-type-1 (j) were from from Santa Cruz Biotechnology.

**
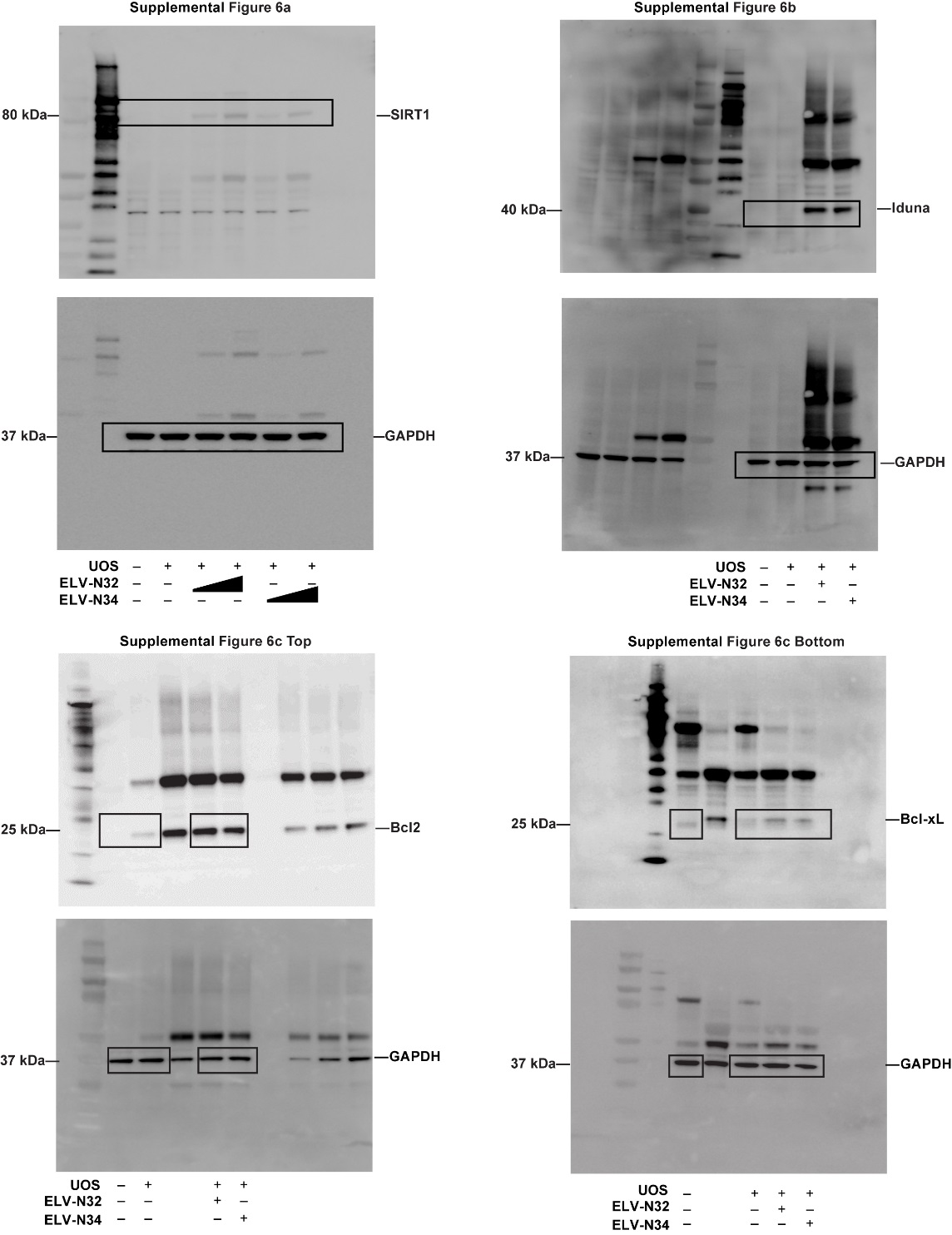
**

**Supplementary Figure 3.** Full length gels of cropped images shown in Figure 6a, b, & c. Western blot analysis representing ELV-N32 and ELV-N34 enhance abundance of pro-homeostatic proteins and decrease the abundance of cell damaging proteins in retinal pigment epithelial (RPE) cell lysates under UOS. The Western blot run, transfer of proteins in nitrocellulose membrane, and protein probing are exactly similar to those described for Figure 3 and in Supplementary Figure 1. The membranes were subjected to probing with primary antibodies SIRT1 (a), Iduna (b), Bcl2/Bcl-xL (c). All blots were stripped and re-probed with anti-GAPDH (Santa Cruz Biotechnology) as a loading control. The bands of interest are indicated by black boxes on the gels. The position of bands for the respective protein molecular weight markers are indicated to the right of the blots.


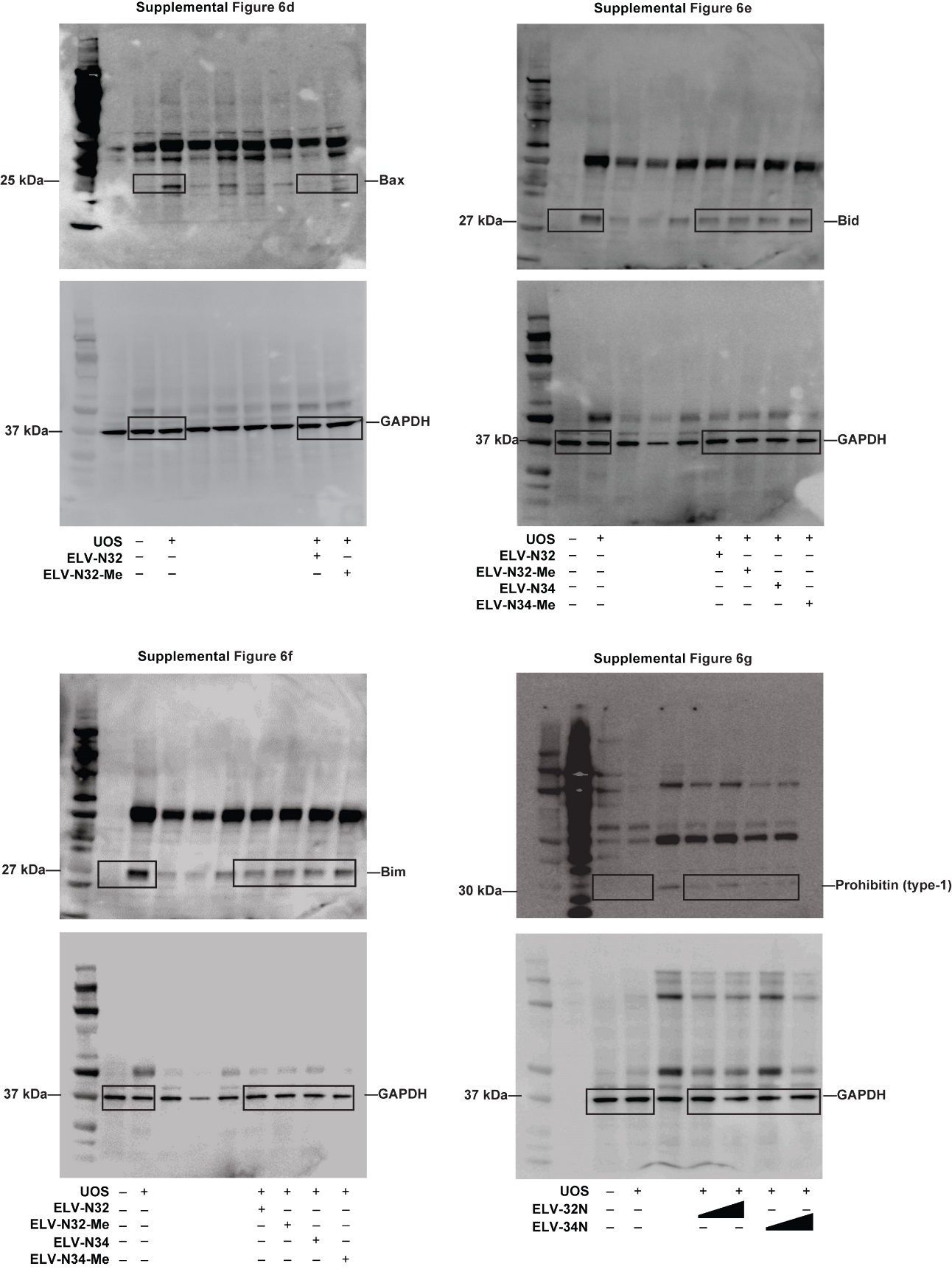


**Supplementary Figure 4.** Full length gels of cropped images shown in Figure 6d, e, f, & g. Methods were the same as described for Figure 6 and in Supplementary Figure 1. The membranes were subjected to probing with primary antibodies Bax (d), Bid (e), Bim (f), and Prohibitin (type 1) (g) from Santa Cruz Biotechnology.
